# Deep ensemble approach for pathogen classification in large-scale images using patch-based training and hyper-parameter optimization

**DOI:** 10.1186/s12859-023-05398-7

**Published:** 2023-07-01

**Authors:** Fareed Ahmad, Muhammad Usman Ghani Khan, Ahsen Tahir, Farhan Masud

**Affiliations:** 1 Department of Computer Science, University of Engineering and Technology, G.T. Road, Lahore, Punjab 54890 Pakistan; 2 Quality Operations Laboratory, Institute of Microbiology, University of Veterinary and Animal Sciences, Outfall road, Lahore, Punjab 54000 Pakistan; 3 Department of Statistics and Computer Science, Faculty of Life Sciences Business Management, University of Veterinary and Animal Sciences, Outfall Road, Lahore, Punjab 54000 Pakistan; 4 Department of Electrical Engineering, University of Engineering and Technology, G.T. road, Lahore, Punjab 54890 Pakistan; 5 National Center of Artificial Intelligence, Al-Khawarizmi Institute of Computer Science, UET, Lahore, Pakistan

**Keywords:** Pathogen classification, Deep learning models, Ensemble learning, Image patching, Feature fusion, Tuning hyper-parameter

## Abstract

Pathogenic bacteria present a major threat to human health, causing various infections and illnesses, and in some cases, even death. The accurate identification of these bacteria is crucial, but it can be challenging due to the similarities between different species and genera. This is where automated classification using convolutional neural network (CNN) models can help, as it can provide more accurate, authentic, and standardized results.In this study, we aimed to create a larger and balanced dataset by image patching and applied different variations of CNN models, including training from scratch, fine-tuning, and weight adjustment, and data augmentation through random rotation, reflection, and translation. The results showed that the best results were achieved through augmentation and fine-tuning of deep models. We also modified existing architectures, such as InceptionV3 and MobileNetV2, to better capture complex features. The robustness of the proposed ensemble model was evaluated using two data splits (7:2:1 and 6:2:2) to see how performance changed as the training data was increased from 10 to 20%. In both cases, the model exhibited exceptional performance. For the 7:2:1 split, the model achieved an accuracy of 99.91%, F-Score of 98.95%, precision of 98.98%, recall of 98.96%, and MCC of 98.92%. For the 6:2:2 split, the model yielded an accuracy of 99.94%, F-Score of 99.28%, precision of 99.31%, recall of 98.96%, and MCC of 99.26%. This demonstrates that automatic classification using the ensemble model can be a valuable tool for diagnostic staff and microbiologists in accurately identifying pathogenic bacteria, which in turn can help control epidemics and minimize their social and economic impact.

## Introduction

Bacteria are ubiquitous in our environment, living on and within us as well as in the atmosphere. Some bacteria are harmless and coexist with other species like animals and birds, while others can cause disease in humans. Approximately 1500 different pathogens are responsible for causing illnesses in humans, including tuberculosis, cholera, pneumonia, diarrhea, plague, and typhoid [1]. These pathogens are not only responsible for significant outbreaks but also cause billions of dollars in economic losses worldwide and disrupt businesses [2].

Accurate and rapid identification of bacterial genera and species is critical in preventing the spread of diseases, especially contagious ones. The current study concentrates on 24 bacterial pathogens, with a focus on the five pathogens (Enterococcus faecium, Acinetobacter baumannii, Pseudomonas aeruginosa, Staphylococcus aureus, and Neisseria gonorrhoeae) that instantly need new therapeutics by the World Health Organization [3]. The DIBaS dataset [4] used in this research includes all of these pathogens. Automated identification of these pathogens using computational approaches is becoming increasingly important. The advent of deep Convolutional Neural Network (CNN) models has the potential to greatly aid in the quick diagnosis, prevention, and treatment of illnesses.

Traditional laboratory techniques for bacterial identification are time-consuming and require expert knowledge and experience. Two key features can help with bacterial recognition: shape and colony structure. Bacterial shape is a distinctive feature that can be identified in an image, but it is difficult to classify bacteria solely based on shape as bacteira can have similar forms or structures. Colony structure, including the shape and size of colonies, is another important characteristic in terms of unique structures and spatial arrangements. However, some bacterial species have morphologically dissimilar cells and can have different forms and sizes, making classification based on shape and colony structure challenging. As a result, specialists may require additional examination using additional microbiological features.

Deep learning models like AlexNet [5], GoogleNet [6], SqueezNet [7], MobileNetV2 [8], and InceptionV3 [9], large-size databases like ImageNet [10], and effective regularization methods “dropout” [11], show improved performance, prediction accuracy, and excellent generalizability to resolve complex computer vision, biological and medical tasks [12]. The benefit of Convolution network’s for image classification is that the network automatically identifies essential features without any human intervention.

However, training deep models with large features on small datasets can result in overfitting. Transfer learning (TL) is a solution to this problem, using the knowledge gained from solving a specific task to solve a different but related problem. The method is effective when combined with augmentation, rigorous hyper-parameter optimization, and appropriate fine-tuning policies. Moreover, deep learning models possess varying architectures, layers, and convolutions, which enable them to learn distinct features from the data. This diversity of features can be leveraged through ensemble learning, a successful approach in computer vision, to integrate distinctive features from different models and achieve consistent and improved predictive performance.

Computer-aided techniques are efficient tools for the classification of bacterial species. During the early days of the research image processing techniques like morphological and geometric properties were applied for bacterial classification. However, recently newly devised machine and deep learning methods are being used in this area of research. Zielinski et al. [4] propose a deep learning approach for bacterial colony classification using Convolutional Neural Networks (CNNs). They provide an open-source image dataset for various bacterial species and use the CNNs for feature extraction. The classification is performed using two traditional machine learning algorithms, Random Forest and Support Vector Machines (SVM). The authors report an impressive accuracy of 97.24% with their proposed approach. However, the limitations of the study include the lack of external testing data to evaluate the generalizability of the design. In recent years different researches have applied DIBaS dataset for training their models. Khalifa et al. [13] developed a deep neural network approach for the classification of bacterial colonies. They used the DIBaS dataset, consisting of 660 images of 33 classes of bacteria, for their study. To overcome the limitation of limited data, the authors applied data augmentation techniques to increase the number of images. The proposed approach was evaluated using a split of 80% of the data for training and 20% for testing, resulting in an accuracy of 98.22%. Muhammed Talo [14] proposed an automated approach for bacterial colony classification by fine-tuning a pre-trained ResNet-50 model on the DIBaS dataset. The study achieved a classification accuracy of 99.12% but used an imbalanced dataset with varying number of images in each class. Additionally, the study did not employ augmentation techniques or perform hyperparameter optimization. Another research by Rujichan et al. [15] proposed a deep learning solution for bacterial colony classification by fine-tuning a MobileNetV2 model. The authors utilized color masking for data preparation and applied various data augmentation techniques to increase the number of training images. The study reported an accuracy of 95.09% for the classification of bacterial colonies. Abd Elaziz et al. [16] applied a novel approach for feature extraction in bacterial colony classification. They used fractional-order orthogonal moments to extract fine features from the images. The authors tested their method on 660 images from the DIBaS dataset and achieved an accuracy of 98.68%. In the study by Gallardo et al. [17], the authors employed a fine-tuned MobileNetV2 model and utilized data augmentation techniques to perform bacterial colony classification. The research was based on the DIBaS dataset, which consisted of imbalanced classes of images. Despite this, the authors were able to achieve an accuracy of 94.22% in their classification results. Satoto et al. [18] proposed an automated classification model for bacterial colonies based on a Convolutional Neural Network (CNN). The study focused on a subset of four classes from the DIBaS dataset and applied data augmentation techniques to increase the diversity of the training data. The model achieved a classification accuracy of 98.59%.

In recent years, the use of deep learning models in the field of bacterial specie classification has increased. However, many of these studies are limited by the small amount of available training data and the absence of testing data to assess the generalizability of the models. Most previous approaches also neglected crucial techniques such as image patching, ensemble learning, data augmentation, and hyperparameter tuning, which have the potential to further enhance the performance of these models. Table 1 gives us an overview of previous approaches presented in the literature.

**Table 1:** A comparison of different deep learning models that applied DIBaS dataset for training

Approach	Technique/ Model	Number of images	Augmentation?	Data split?	Testing data?	Image patching?	Balanced dataset?	Ensemble model?	Hyper-parameter Tunings?
[4]	VGG16, SVM	660	$\times$	50:50	$\times$	$\times$	$\checkmark$	$\times$	$\times$
[19]	VggNet, AlexNet	35600	$\times$	80:20	$\times$	$\checkmark$	$\times$	$\times$	$\times$
[20]	BoW,SVM	200	$\times$	70:30	$\times$	$\times$	$\checkmark$	$\times$	$\times$
[14]	ResNet50	689	$\times$	80:20	$\times$	$\times$	$\times$	$\times$	$\times$
[16]	MFrLFMs, SSATLBO	660	$\times$	80:20	$\times$	$\times$	$\checkmark$	$\times$	$\times$
[17]	MobileNetV2	669	$\checkmark$	80:10:10	$\checkmark$	$\times$	$\times$	$\times$	$\checkmark$
[18]	CNN	1000	$\checkmark$	80:20	$\times$	$\checkmark$	$\checkmark$	$\times$	$\times$
[21]	VGG16	660	$\checkmark$	80:10:10	$\checkmark$	$\times$	$\times$	$\times$	$\times$

The Images column describes the number of images in the dataset. Augmentation? column elaborate whether the researcher applies data augmentation. Data split? describes the ratio in which the dataset is divided. Testing data? means whether testing data was kept for checking models peformance. Similarly the Image Patching? column indicates whether the large-scale images were divided into smaller images. The column Balance Dataset? displays if the approach uses a dataset with equal number of image instances in each class. The column Ensemble Model? reflects whether the technique applies ensemble learning. The last column Hyper-parameter Tuning describes whether the research uses various variations of Learning rate, Batch size,and Epochs etc

**Fig. 1:**
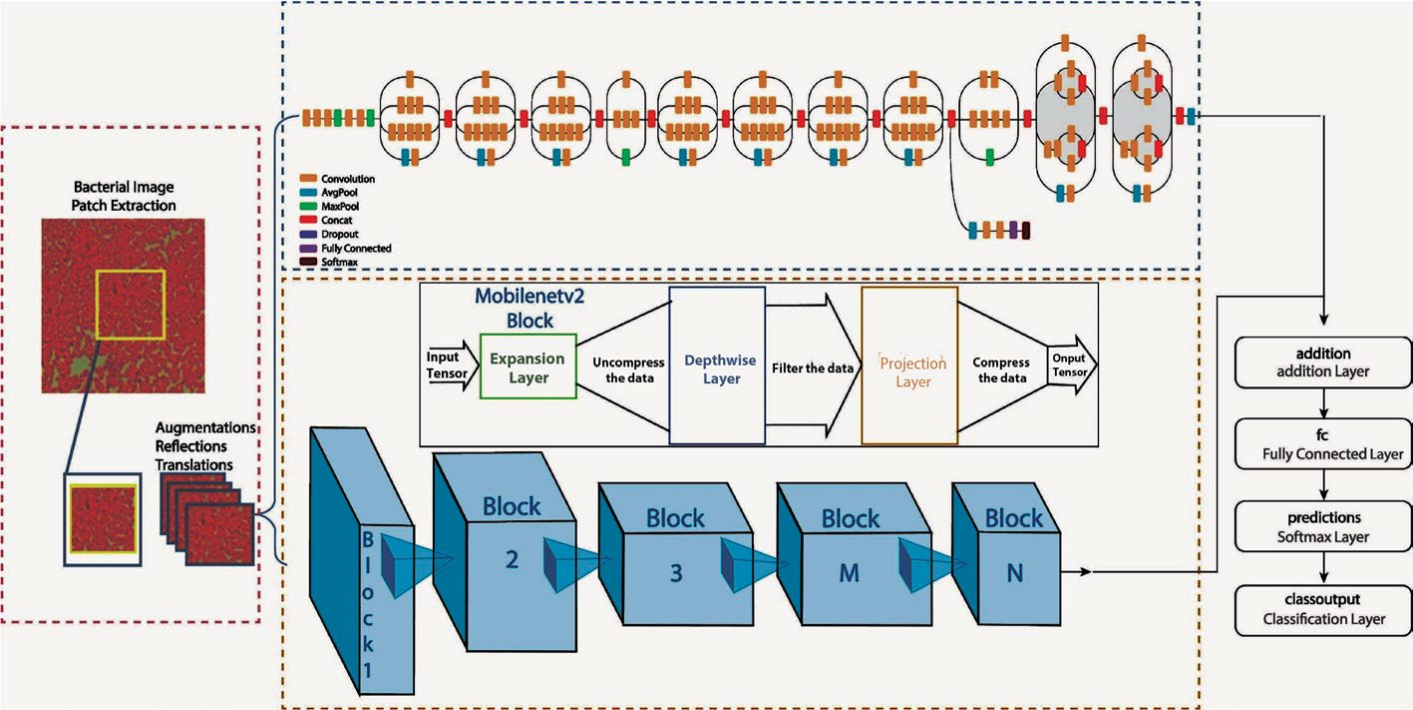
Various phases of our proposed method

The gap in the existing studies that motivated this research is the need for a more robust and accurate classification model for bacterial species. This is particularly important in the classification of pathogenic bacteria, as incorrect classification could have serious implications for public health. To address this gap, this research presents an extensive and balanced dataset prepared by segmenting high-scale images using image patching, and each class of the dataset now consists of 320 images. The bacterial images are trained using a combination of transfer learning, fine-tuning, hyper-parameter tuning, and augmentation strategies applied on pre-trained models. The research also introduces a deep model, which combines the distinctive features of InceptionV3 and MobileNetV2, using an ensemble learning technique, as illustrated in Fig. 1.

Our research contribution can be summarized as follows:

1. An extensive and balanced dataset was prepared through image patching.
2. An ensemble learning design combining InceptionV3 and MobileNetV2 architectures with additional dense layers and a dropout layer was proposed.
3. The research blended transfer learning, fine-tuning, hyper-parameter tuning, and augmentation in one design.
4. The focus of the research was specifically on the classification of pathogenic bacteria.
5. Two data splits were applied to evaluate the robustness of the ensemble model with increased training data from 10 to 20%.

## Materials and methods

In our study, we used two state-of-the-art deep learning architectures, MobileNetV2 and InceptionV3, to classify bacterial colonies based on their gram-stain images. The architectural differences between MobileNetV2 and InceptionV3 allow them to capture distinct features from the data. MobileNetV2 focuses on efficiently capturing spatial details using depthwise separable convolutions, making it effective at capturing fine-grained features and patterns [8]. In contrast, InceptionV3 utilizes factorized inception blocks to gather a wide range of feature patterns at varying scales [9].

These architectural variances lead to differences in the types of features learned by each model. MobileNetV2 excels at capturing intricate textures, edges, and local patterns, while InceptionV3 excels at capturing more global features, such as object shapes or larger contextual information [8, 9]. By combining the outputs of these models in the ensemble, we leverage their complementary learned features. The ensemble model benefits from the diverse representations captured by each model, resulting in improved performance by capturing a broader range of discriminative features from the bacterial colony images.

**Fig. 2:**
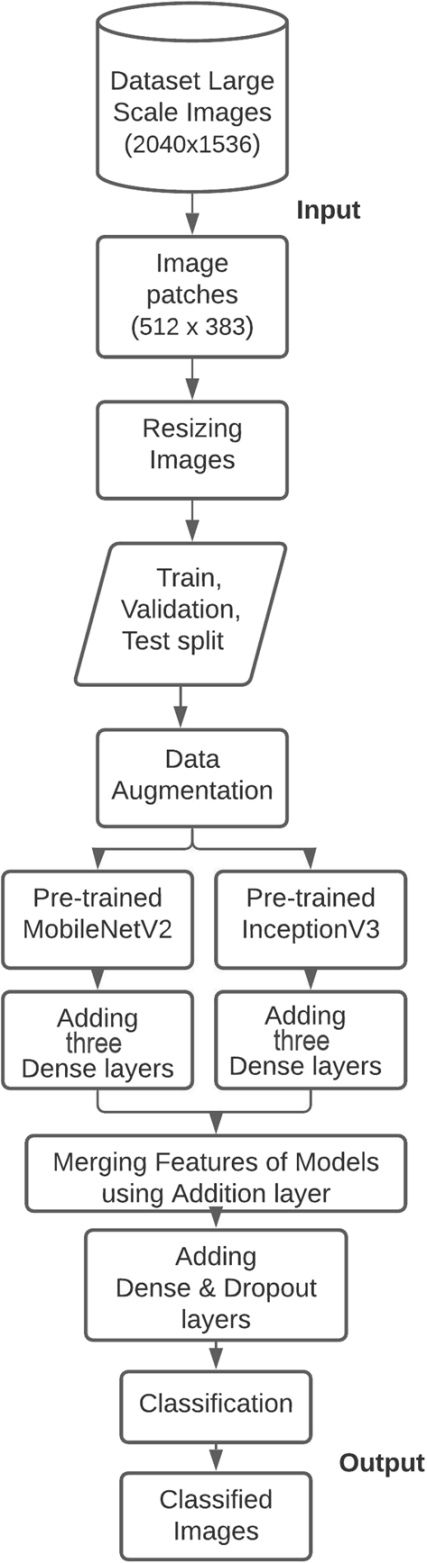
Flowchart of different stages of our deep ensemble model

A CNN model for merging features of pre-trained models with the help of ensemble learning is given in the Fig. 2. The presented design comprises of the following steps: (i) Segmenting a large-scale image into patches (ii) Image resizing (iii) Dataset splitting (iv) Data augmentation (iv) Embedding pre-trained MobileNetV2 and InceptionV3 models to the framework (v) Adding Dense layers to these models (vi) Merging features of these models using addition layer (vii) Adding some dropout and dense layers and eventually applying classification on the suggested ensemble design.

Algorithm 1 portrays the pseudocode of the suggested deep ensemble design. Initially, large scale gram-stained bacterial images are segmented into smaller patches. These segmented images are then resized to meet the input size requirement of the deep learning models. The resized images are then split into train, validation, and test datasets, and data augmentation techniques are applied to produce an augmented dataset. Two pre-trained deep models, MobileNetV2 and InceptionV3, are embedded into the ensemble model and dense layers are added before merging their features. Finally, dropout and dense layers are added to further enhance the quality of the model, and then classification is performed to obtain the class labels of the 24 categories of pathogenic bacterial images as output.

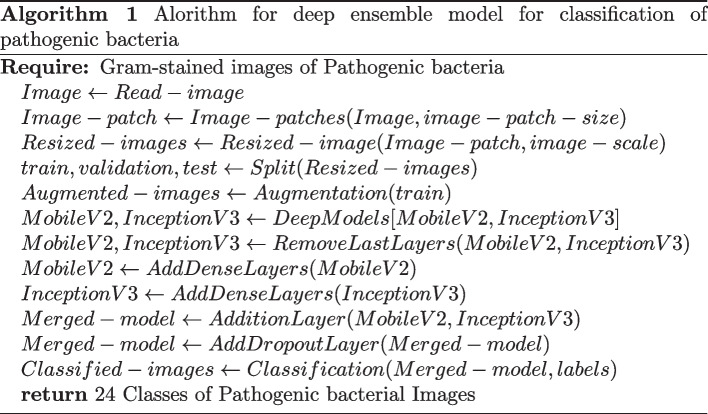



### Dataset details and augmentation

In our research, we utilied the DIBaS dataset [4] that comprises of annotated, high resolution, and microscopic images from thirty three species of bacteria. Out of these, 24 pathogenic species are selected, which are present in various environment’s and cause different diseases [22]. Each strain consists of 20 images of a specific class. Various augmentation techniques (random vertical translation, horizontal reflection, and translation) are applied to produce an augmented dataset. For experimentation, we divide the dataset into 3 sets, i.e., train, validation, and test data, with split ratio’s of 7:2:1 and 6:2:2.

### Patch extraction and data preprocessing

**Fig. 3:**
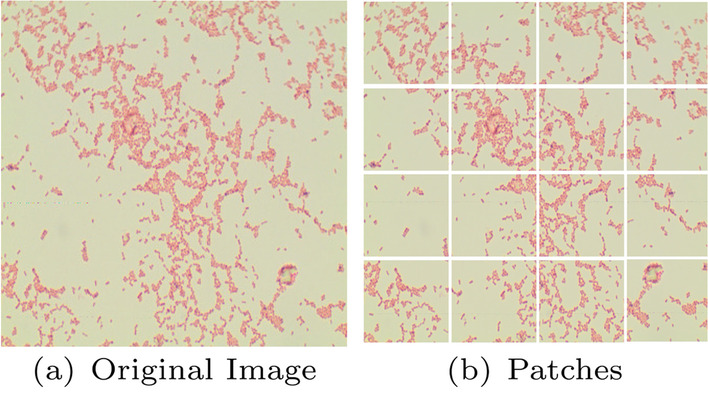
Original Image of size 2040x1536 and its Patches of 512 x 383

The High dimensional images (2040 x 1536) consume a subsequent amount of memory. So, we segment each image into 16 patches of dimension i.e. (512 x 383), as shown in Fig. 3. Hence, now we have 320 bacterial images relating to each category. The patches with a white background and no significant information are removed. In the research, we have assessed different deep learning models. Each model’s input size is different like InceptionV3 has an input image size of 299-by-299, and MobileNetV2 has dimensions of 224-by-224. So, the images need to be further adjusted to meet the input requirement of the models.

### Hyper-parameters tuning

We evaluated the effect of different hyper-parameter optimizations on the model performance, such as the learning rate, batch size, and epochs. In addition to the proposed model, we also assessed five CNN models and implemented fine-tuning using different parameters like learning rate, batch size, and number of epochs, etc. Table 2 shows the details of hyper-parameters utilized to optimize these models during the training phase.

**Table 2:** The details of hyper-parameters applied for various CNN models

CNN model	Batch-size	Learning-rate
AlexNet	48,24,16	8e$-$7,1e$-$8,1e$-$7,6e$-$7,1e$-$5,1e$-$6,1e$-$10
SqueezeNet	48,24,16	1e$-$8, 1e$-$7, 1e$-$6, 9e$-$5
GoogleNet	48,24,16	1e$-$8, 6e$-$7, 1e$-$7, 9e$-$6, 1e$-$6, 1e$-$5
MobileNetV2	24,16,8	9e$-$6, 3e$-$6,5e$-$6,1e$-$7,8e$-$5,1e$-$6
InceptionV3	48,24,16	8e$-$5,1e$-$6,9e$-$6,1e$-$5,1e$-$7
Our ensemble model (MobileNetV2 +InceptionV3 )	24,16,8	4e-7, 1e-7, 1e-5, 1e-6

### Convolution neural networks

The motivation for using Convolutional Neural Network (CNN) models in computer vision tasks is due to their ability to handle large amounts of data and learn and extract meaningful features from images automatically that improve the accuracy of predictions. Unlike traditional machine learning models that require hand-crafted features, CNNs can learn hierarchical representations of images that capture complex relationships between pixels. They normally consists of three components: convolution, pooling, and dense layers. During a convolution operation, various filters are applied to extract features (feature map) from the image, by which their spatial information can be conserved. The pooling method is also known as subsampling, which is used to decrease the dimensionality of feature maps and also to pick the most vital feature from the convolution process. Dense layers, also known as fully connected layers, play a crucial role in making final predictions by mapping high-level features from convolution and pooling layers to output classes or labels. They are responsible for capturing complex relationships and patterns in the data to provide accurate predictions.

The training of CNN’s starts from the first input layer and goes up till the final layer. The error is back-propagated from the last classification layer to the initial convolutional layer. If *n* is a neuron in layer *h*, which recieves input from a neuron *m* of layer ${h-1}$,the sum input $In_{n}^{h}$ can be computed as follows:

1 $$\begin{aligned} In_{n}^{h}=\sum _{m=1}^{n} W_{nm}^{h} x_{m} + b_{n} \end{aligned}$$

where $b_{n}$ and $W_{nm}^{h}$ are the bias term and weight vector of the $h^{th}$ layer respectively.The output of the $h^{th}$ layer can be computed by the ReLU function as:

2 $$\begin{aligned} Out_{n}^{h}=max(0, In_{n}^{h}) \end{aligned}$$

The connections in the fully connected and convolution layers utilize Eqs. 1 and  2 to calculate the inputs/outputs. For more details on working of CNNs, the researchers may refer to [23]. In this section, we will discuss two popular CNN models, MobileNet and InceptionNet, and their motivations.

### Pre-trained architectures

Different pre-trained models were utilized in our research, including MobileNetV2 and InceptionV3. These models are pre-trained on the ImageNet dataset, which consists of a vast collection of images across multiple classes. The pre-training process enables these models to learn generic features from the ImageNet dataset [10], which can then be fine-tuned for our specific task of classifying pathogenic bacteria. The following sub-sections provide a detailed description of these pre-trained models and their architectures.

#### AlexNet

**Fig. 4:**
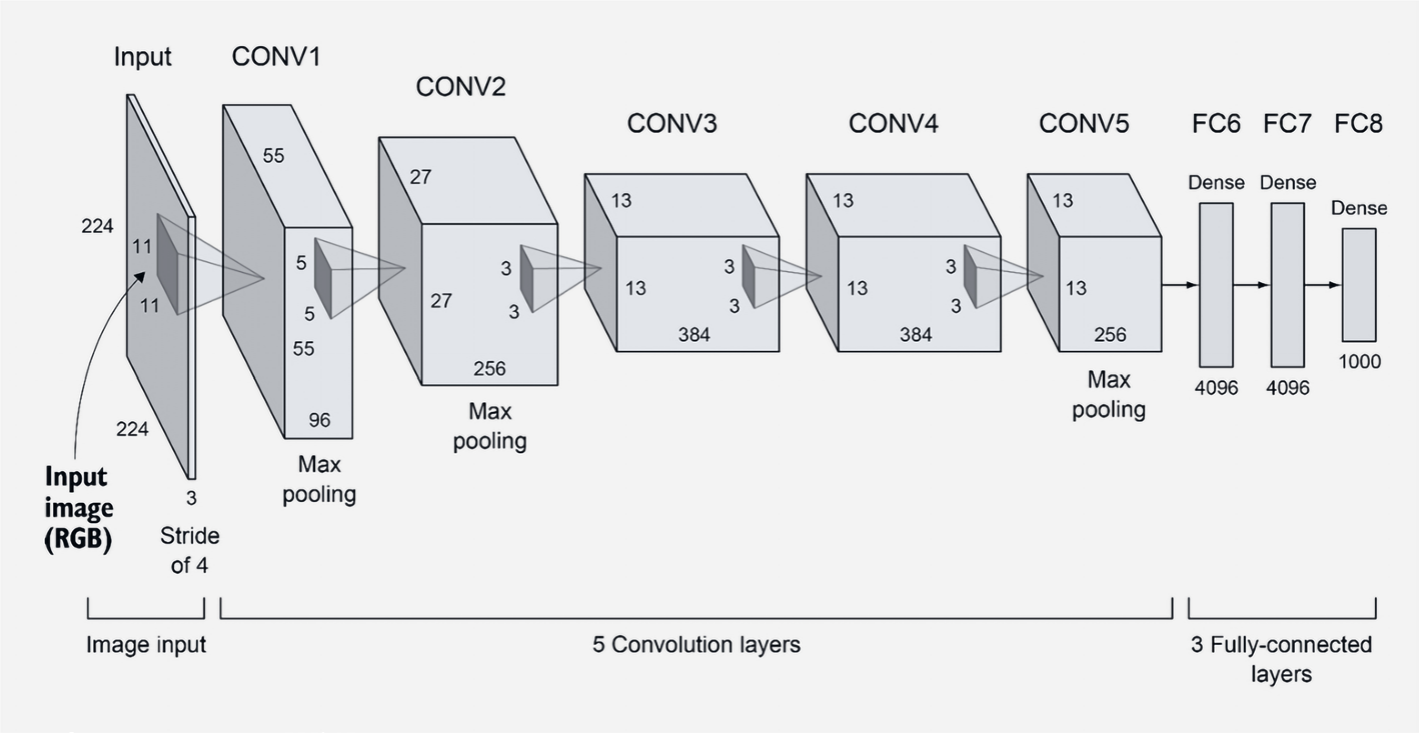
AlexNet architecture

The AlexNet was the victorious model in the 2012 ImageNet competition, primarily utilized for image classification. It is an expanded and deeper version of LeNet that includes the fundamental components of CNNs and serves as the basis for other deep learning architectures. While preserving the original design, it has added features such as LRN, dropout, and ReLU. The key finding from the study was that the model’s impressive performance was largely due to its depth, although this came at the cost of increased computational demands during training, which were made feasible through the use of GPUs. The model consists of 5 convolutional and 3 fully connected layers, as illustrated in the Fig. 4.

#### SqueezeNet

**Fig. 5:**
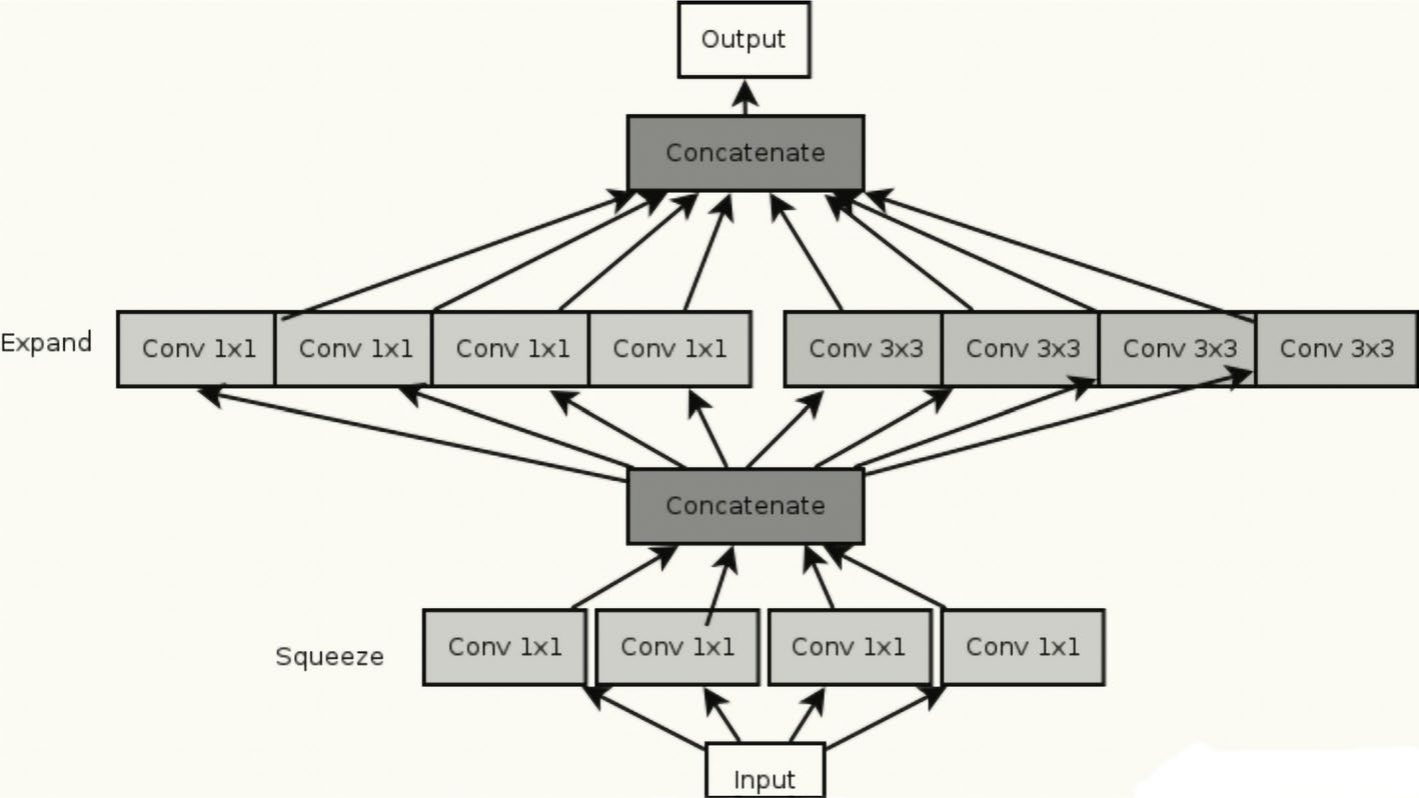
SqueezeNet fire module

The Squeeznet model was developed by researchers from Stanford, Berkeley, and DeepScale with the aim of creating a smaller CNN with fewer parameters that would consume less memory and could be efficiently transmitted over a computer network. The foundation of this deep learning architecture is the fire module, which consists of a convolution layer with 1x1 filters that feed into an expand layer that has both 1x1 and 3x3 convolution filters, as depicted in the Fig. 5.

#### GoogleNet

Google introduced the inception structure called GoogLeNet, which was the best performer at the 2014 ImageNet competition. While constructing a Convolution Network you have to pick 1 x 1, 3 x 3, 5 x 5, convolution layer, or pooling layer. The GoogleNet has them all in its inception module. It makes architecture complicated but works remarkably well.

#### MobileNetV2

MobileNetV2 is a computationally efficient CNN model that is well-suited for mobile and embedded systems. Its design focuses on reducing the number of parameters, computations, and memory usage while still achieving good performance on image classification tasks. This makes it ideal for deployment on resource-constrained devices such as smartphones, which have limited computational resources. MobileNetV2 is quite similar to MobileNetV1, which offers a depthwise separable convolutional layer that reduces the size and complexity of the model. It also includes a useful module with an inverted residual block with bottlenecking features. It has a drastically lesser parameter than the initial MobileNetV1 design [24]. Figure 1 illustrates the MobileNetV2 block.

#### InceptionV3

**Fig. 6:**
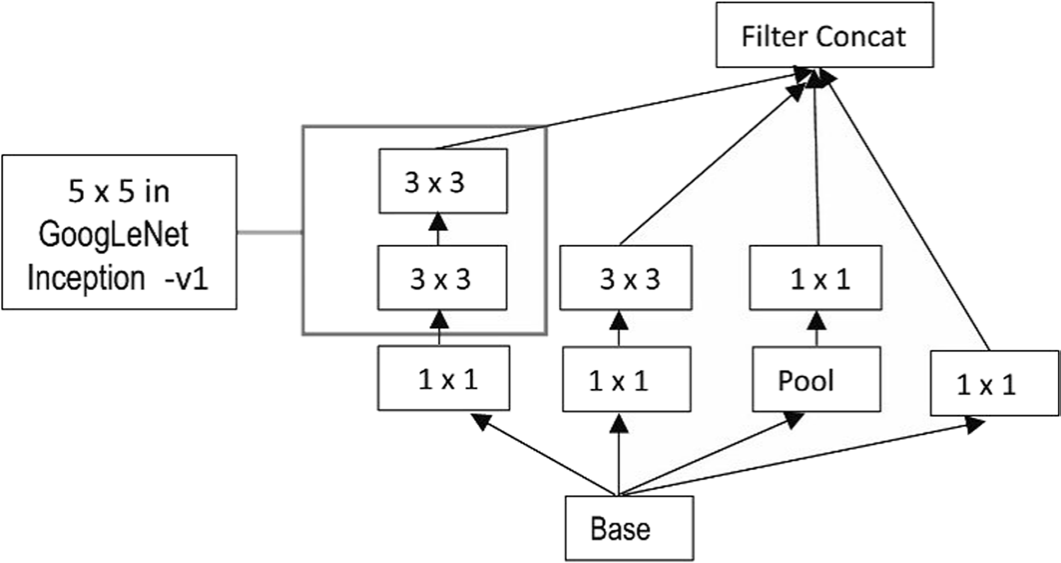
InceptionV3 Module

InceptionNet is a flexible and scalable CNN model that is designed for a wide range of computer vision tasks. It features an Inception module that allows for multiple parallel convolutions of different kernel sizes, which can extract features from an image at multiple scales. This makes InceptionNet well-suited for tasks such as object detection, semantic segmentation, and image classification. Additionally, its scalability makes it possible to adapt the model to different data sets and computational resources. Like MobileNetV2, it strikes a balance between accuracy and computational cost, making it a good choice for our study. The inceptionV3 model is an updated form of inceptionV2 that attains excellent results on image recognition challenges by removing 5 $\times$ 5 convolutions and including preferably two more 3 $\times$ 3 convolution layers. The model restricts overfitting and tend to achieve label smoothing. It also factorizes a 7 $\times$ 7 convolution layer and combines different CNN layers after normalization, rendering greater accuracy with limited computation complexity, as shown in Fig. 6.

### Ensemble classification

These CNN’s are non-linear architectures that learn complex relations from input data through stochastic optimizations and error back-propagation, which makes them very responsive to weight initializations and the noise present in the dataset. Ensemble learning resolves these issues by training several models and joining their predictions or features. In this technique, model shortages are balanced by the predictions of the other architectures. Merged predictions produce better results than any single model [25]. Ensemble learning techniques reduce variance error, enhance performance, and generalizability of models.

### Performace metrics

To evaluate the performance of the different deep models in comparison to the proposed methodology, multiple performance measures are employed, including precision, recall, F-Score, and Matthews Correlation Coefficient (MCC), in addition to accuracy. This comprehensive set of metrics provides a more complete assessment of the model’s effectiveness, as accuracy alone may not sufficiently capture its performance [26].

By considering precision, recall, F-Score, and MCC in conjunction with accuracy, we gain insights into different aspects of the model’s predictive capabilities, such as its ability to correctly identify positive instances (precision), capture all relevant positive instances (recall), achieve a balance between precision and recall (F-Score), and provide an overall correlation measure (MCC). Together, these performance measures offer a comprehensive evaluation of the deep models’ effectiveness in the context of the proposed methodology. The computation of these metrics involves the following equations, where TP represents true positives, TN represents true negatives, FP represents false positives, and FN represents false negatives:

3 $$\begin{aligned} \text{ Accuracy } = \frac{\textrm{TN}+\textrm{TP}}{\textrm{FP}+\textrm{TP}+\textrm{FN}+\textrm{TN}} \end{aligned}$$

4 $$\begin{aligned} \text{ Precision }= \frac{\textrm{TP}}{\textrm{FP}+\textrm{TP}} \text{, } \end{aligned}$$

5 $$\begin{aligned} \text{ Recall }= \frac{\textrm{TP}}{\textrm{FN}+\textrm{TP}} \end{aligned}$$

6 $$\begin{aligned} \text{ FScore }= \frac{2 \times \text{ Precision } \times \text{ Recall } }{ \text{ Precision } + \text{ Recall } } \end{aligned}$$

7 $$\begin{aligned} \textrm{MCC}= \frac{T P \times T N-F P \times F N}{\sqrt{(T P+F P)(T P+F N)(T N+F P)(T N+F N)}} \end{aligned}$$

### Model training strategies

In the Results section, various experimental Strategies were adopted to demonstrate the effects of fine-tuning and augmentation on deep learning models with and without pre-trained weights from the ImageNet dataset. These strategies are as follows:

#### Training from the beginning

In this approach, deep models are trained from scratch on the target dataset without applying any parameter-tuning or utilizing any previously learned weights from the ImageNet dataset.

#### Parameter-tuning without pre-trained weights

In this strategy, various parameters such as learning rate, batch size, etc., of deep models are tuned on the target dataset without utilizing any previously learned weights from the ImageNet dataset.

#### Fine-tuned with pre-trained weights when all layers unfrozen

In this technique, deep models are fine-tuned on the target dataset using previously learned weights from the ImageNet dataset. However, the weights of the layers are not frozen, and they are updated during the training process.

#### Augmented and fine-tuned with pre-trained weights when all layers are unfrozen

In this strategy, deep learning models are fine-tuned on the target dataset, and various augmentation techniques are applied to increase the size of the training data. The models utilize previously learned weights from the ImageNet dataset, and the weights are updated as the models learn during training.

## Results

Initially, we analyze the performance of the CNN models in four different scenarios. When the deep learning models are: (i) trained from scratch (ii) Parameter-tuning without pre-trained weights (iii) fine-tuned with pre-trained weights when all layers un-frozen, and (iv) augmented and fine-tuned with pre-trained weights when all layers are unfrozen. For every approach, the outcomes of loss and accuracy of models are shown in Tables 3 and 4. By looking at it, we can infer the following conclusions:

1. The results depicit that shallow models produce substantially better results than deeper models for stratergy (i) and (ii) for small-size dataset like DIBaS. Specially in case of (ii) we can see that only tuning model parameters like learning-rate, batch size etc. without any pre-trained weights from ImageNet show significant better results in shallower models as compared to deeper models because these deeper models need substaintially larger number of parameters to train as compared to shallow models, as reflected by the results in Tables 3 and 4.
2. Applying fine-tuning on a pre-trained model is a highly efficient transfer learning approach for image classification tasks. The results show improvement in the accuracy of all CNN models fine-tuned on the primary dataset.
3. Augmentation is extremely beneficial for enriching a design’s performance, notably when the dataset is inadequate. The deep learning designs, together with traditional augmentation procedures, can help in achieving excellent results. The results display that the strategies where augmentation was applied show a 1–6% enhancement in test accuracy, and the reduction in loss ranges from 19 to 91% over prior fine-tuned deep models without augmentation.
4. The two best-performing models, MobileNetV2 and InceptionV3, were selected for feature fusion in our ensemble model I. The results showed that these models produced a test accuracy of 97.79% and 97.92% and a minimum loss of 0.0212 and 0.0446, respectively.
5. The proposed ensemble model I and II, with a split ratio of 7:2:1, produces a validation accuracy and loss of 99.41% and 0.0275, respectively, for model I; and 97.14% and 0.0953, respectively, for model II. The test results for model I include an accuracy of 99.91%, F-Score of 98.95%, precision of 98.98%, recall of 98.96%, and MCC of 98.92%. For model II, the test results are an accuracy of 99.79%, F-Score of 97.52%, precision of 97.60%, recall of 97.53%, and MCC of 97.44%.
6. Similarly, the ensemble model I and II, with a split ratio of 6:2:2, produces a validation accuracy and loss of 98.96% and 0.0284, respectively, for model I; and 97.72% and 0.3087, respectively, for model II. The test results for model I include an accuracy of 99.94%, F-Score of 99.28%, precision of 99.31%, recall of 98.96%, and MCC of 99.26%. For model II, the test results are an accuracy of 99.85%, F-Score of 98.24%, precision of 98.30%, recall of 98.24%, and MCC of 98.18%.
7. These results indicate that both ensemble models I and II demonstrate significant improvements over their respective base models, InceptionV3 and MobileNetV3 for model I, and GoogleNet and SqueezeNet for model II. The performance enhancements are consistently observed across the different evaluation metrics, highlighting the effectiveness of the ensemble approach.

**Fig. 7:**
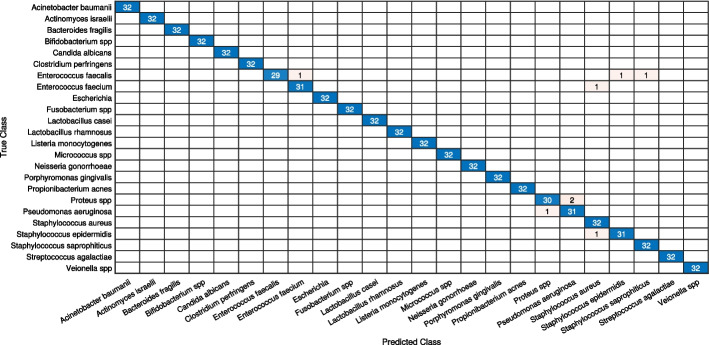
Confusion matrix of ensemble learning model for 10% test data

**Fig. 8:**
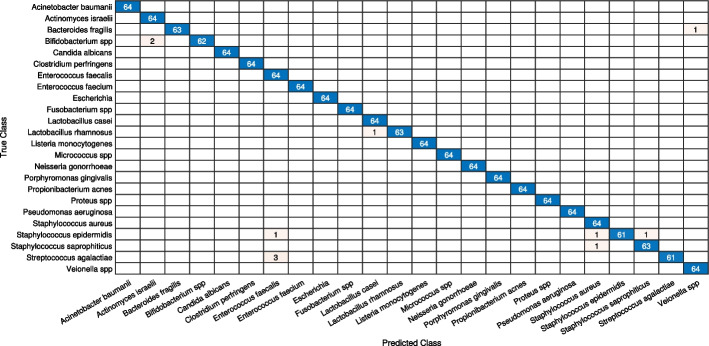
Confusion matrix of ensemble learning model for 20% test data

Furthermore, we investigated the performance of ensemble model I on the test dataset, focusing on two different aspects: i) classification of bacteria from similar genera, and ii) classification of bacteria from dissimilar genera. Based on the confusion matrices shown in Figs. 7 and  8, we draw the following conclusions:

1. There are 8 misclassification’s in Model I with 10% test data and only 9 misclassification’s for model with 20% test data.
2. The Model I exhibitions exceptional result for similar and dissimilar genera.
3. Additionally, for 10% test data, there are 6 misclassifications for different genera and 2 misclassifications for the same genera. For 20% test data, there are 6 misclassifications for different genera and 3 misclassifications for the same genera.
  These findings provide valuable insights into the performance of ensemble model I when classifying bacteria from similar and dissimilar genera.

**Table 3:** Comparative analysis of the Deep Ensemble design with various Deep learning architectures with a data split of 70:20:10.

Model	Methods	Validation-loss	Validation-accuracy	Test-accuracy
Proposed model I (MobileNetV2+InceptionV3)	FT on ADS-ALUF	0.0275	99.41	99.91
Proposed model II (GoogleNet+SqueezeNet)	FT on ADS-ALUF	0.0953	97.14	99.79
AlexNet	Trained on ODS-NPTW	4.1254	13.53	11.98
	PT on ODS-NPTW	0.8999	75.47	76.43
	FT on ODS-PTW	0.2375	93.10	93.10
	FT on ADS-ALUF	0.1056	96.23	96.09
SqueezeNet	Trained on ODS-NPTW	3.1779	6.51	8.07
	PT on ODS-NPTW	0.3370	79.83	82.16
	FT on ODS-PTW	0.3102	87.51	87.89
	FT on ADS-ALUF	0.2614	94.60	93.62
GoogleNet	Trained on ODS-NPTW	4.1299	2.93	1.95
	PT on ODS-NPTW	0.3924	87.77	88.41
	FT on ODS-PTW	0.5174	90.83	90.63
	FT on ADS-ALUF	0.2897	95.64	93.75
MobileNetV2	Trained on ODS-NPTW	3.1169	5.27	5.86
	PT on ODS-NPTW	2.1408	44.05	39.84
	FT on ODS-PTW	0.2569	93.10	92.45
	FT on ADS-ALUF	0.0212	97.85	97.79
InceptionV3	Trained on ODS-NPTW	3.4025	1.69	0.91
	PT on ODS-NPTW	2.4302	56.93	57.03
	FT on ODS-PTW	0.4128	96.62	96.74
	FT on ADS-ALUF	0.0446	98.39	97.92

Here, ADS, ODS, PTW, NPTW, ALUF, PT, FT stands for augmented dataset, original dataset, pre-trained weights, no pre-trained weights, all layers un-frozen, parameter-tuning, fine-tuned

**Table 4:** Comparative analysis of the Deep Ensemble design with various deep learning architectures with a data split of 60:20:20.

Model	Methods	Validation-loss	Validation-accuracy	Test-accuracy
Our proposed model I (MobileNetV2+InceptionV3)	FT on ADS-ALUF	0.0284	98.96	99.94
Proposed model II (GoogleNet+SqueezeNet)	FT on ADS-ALUF	0.3087	97.72	99.85
AlexNet	Trained on ODS-NPTW	5.4068	8.07	8.46
	PT on ODS-NPTW	0.6312	74.37	74.87
	FT on ODS-PTW	0.3788	95.64	94.14
	FT on ADS-ALUF	0.3230	94.93	94.86
SqueezeNet	Trained on ODS-NPTW	3.1764	5.86	5.53
	PT on ODS-NPTW	0.3806	79.38	79.62
	FT on ODS-PTW	0.2673	86.47	86.33
	FT on ADS-ALUF	0.0646	93.43	92.90
GoogleNet	Trained on ODS-NPTW	3.2394	4.16	4.17
	PT on ODS-NPTW	0.3559	88.68	88.35
	FT on ODS-PTW	0.3937	91.35	90.76
	FT on ADS-ALUF	0.2774	95.71	95.83
MobileNetV2	Trained on ODS-NPTW	3.3572	4.16	4.10
	PT on ODS-NPTW	1.8974	43.98	41.73
	FT on ODS-PTW	0.4196	93.62	94.73
	FT on ADS-ALUF	0.0879	95.95	96.16
InceptionV3	Trained on ODS-NPTW	3.3490	4.03	4.23
	PT on ODS-NPTW	2.3959	58.82	57.94
	FT on ODS-PTW	0.4737	95.51	96.06
	FT on ADS-ALUF	0.2032	97.14	96.68

Here, ADS, ODS, PTW, NPTW, ALUF, PT, FT stands for augmented dataset, original dataset, pre-trained weights, no pre-trained weights, all layers un-frozen, parameter-tuning, fine-tuned

Overfitting can be an important matter, specially with small datasets. The model may achieve excellent accuracy, but when analyzed for unseen real-life examples, it may not generalize well for new examples. So, a vital issue to examine if there is overfitting or the design has generalized well for examples provided during training of the model. We access it by estimating the gap within validation and training curves, wider the gap among them higher the overfitting.

**Fig. 9:**
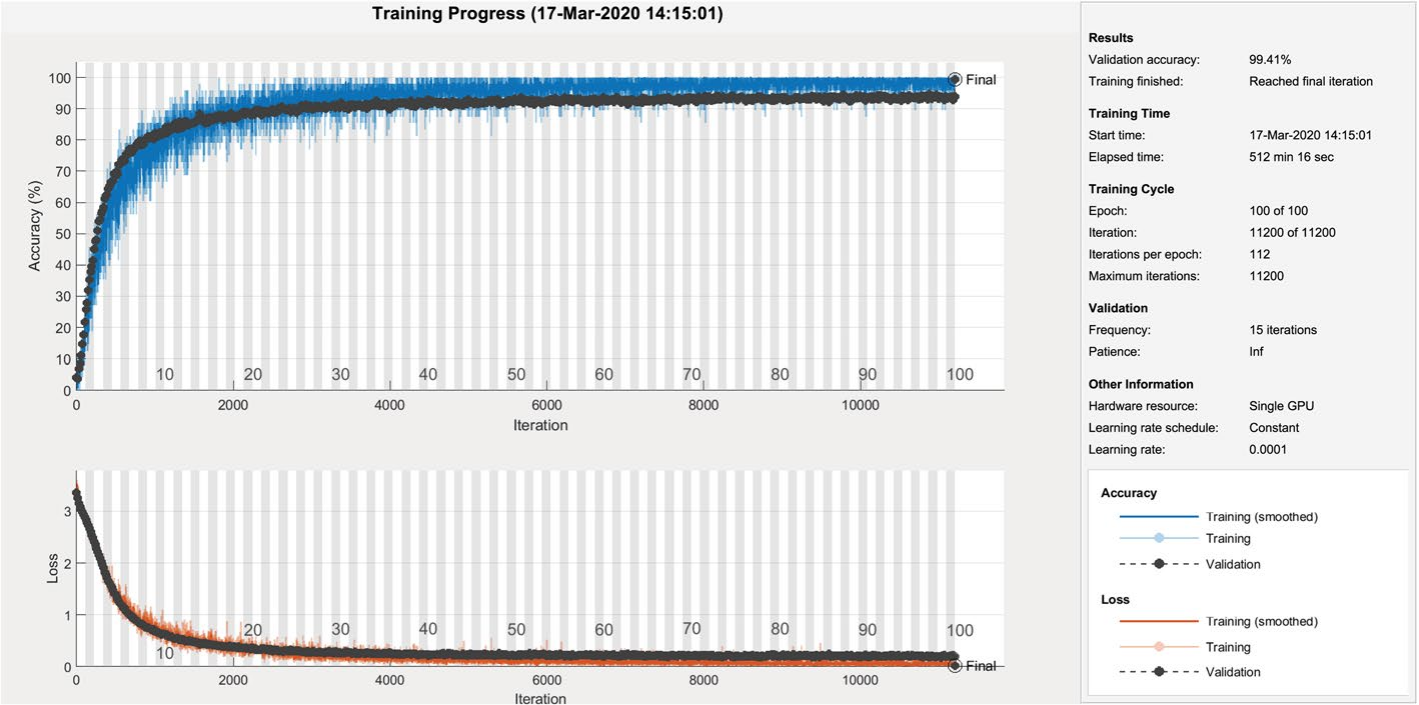
Model Performance Curves for train and validation accuracy (blue, black dotted lines) and train and validation loss (orange, black dotted lines) of Deep Ensemble model, for the Classification of Bacterial pathogens using DIBaS dataset

Figure 9 depicts that the validation and training curves either overlap or move alongside each other without any significant gap, which shows that the model has generalized accurately without any overfitting.

### A comparison against previous works on bacterial colony classification

In order to provide a comprehensive comparison between our ensemble model and other state-of-the-art classifiers, we conducted additional experiments using a common dataset consisting of 660 images representing 33 bacterial classes. This ensures that the number of bacterial species is consistent across all models, eliminating any potential bias caused by variations in class distribution. To ensure a fair evaluation, we adopted the same train, validation, and test split as utilized in our proposed model. Furthermore, we carefully fine-tuned the compared models based on the specified experimental settings provided by the authors. This included factors such as the number of epochs, batch size, learning rate, and any additional dense or dropout layers added to the model. By following this approach, we aim to provide a comprehensive and unbiased comparison between our ensemble model and the other classifiers, enabling a more accurate assessment of their respective performances. While assessing the performance indicators in Table 5, the suggested ensemble models display better results for all the performace metrics applied for assissing the model performance.

**Table 5:** A comparison of our approach with previous deep learning methodologies for bacterial classification

Approach	Number of images	Augmentation	Data split	Loss	Accuracy	Precision	Recall	FScore	MCC
Proposed model 1	660	$\checkmark$	7:2:1	0.2674	99.91	98.98	98.48	98.38	98.52
Proposed model 2	660	$\checkmark$	7:2:1	0.0431	99.82	97.98	96.97	96.77	97.04
ResNet-50 [14]	660	$\checkmark$	7:2:1	0.0155	99.72	–	95.45	94.34	–
MobileNetV2 [17]	660	$\checkmark$	7:2:1	3.0262	95.04	–	18.18	11.64	–
VGG16 [21]	660	$\checkmark$	7:2:1	3.5460	94.58	–	10.61	5.82	–

## Discussion

Deep and machine learning procedures are generating outstanding results in the area of pathogen classification [27], bacterial identification [4], COVID19 [25]. They can help in the immediate and reliable diagnosis of diseases.

Our study reveals that transfer learning can significantly improve current modes of identification of bacterial images while also independently yielding exceptional results for small datasets. Researches [28] reveal that transfer learning can generate outstanding performance, especially for small datasets [29].

Fine-tuning helps models to converge quicker and acquires refine and insightful features that capture intricate image details  [30], as is visible in our approach. It is also effective and efficient technique for diverse classification tasks in the biological domain [31].

Image patching preserves important local details that would otherwise be lost due to down-scaling [30]. Researches have elaborated that image patching help increase dataset size [32] and preserve essential local image details [30].

We also applied augmentation to our model, which produced excellent results as compared to other models. Studies show that augmentation boosts performance and generates a generalized model without overfitting [29].

In our ensemble learning approach, we utilized both MobileNetV2 and InceptionV3, where MobileNetV2 is an improvement of Resnet’s residual block and faster than MobileNetV1 due to its efficient design  [33]. Additionally, InceptionV3 demonstrated remarkable performance in skin cancer diagnosis and applied factorized inception blocks for the accumulation of low-level to high-level feature patterns through smaller and larger convolutions [34]. The combination of these unique features learned by the two models resulted in improved performance compared to using a single model, as the diverse architecture and layers allowed them to learn distinctive features, providing a more comprehensive understanding of the data. Our work shows that suggest that ensemble models acquire beneficial features and generate better performance than individual models, as in Tables 3 and 4. Ensemble models have achieved excellent results in image classification tasks in multiple fields like radiology images [35], and histopathology images [36].

Current improvements in computer-vision are usually dependent on extensive, annotated data, which are not conveniently accessible in the biological field. Hence, our proposed model can be extremely helpful for environments where the dataset is limited and may continue to be especially beneficial in the days to come for the automatic diagnosis of disease-causing bacteria.

## Conclusion

The work presents a classification technique for pathogenic bacteria, which leverages the advantages of ensemble learning, image patching, transfer learning, fine-tuning, and data augmentation. Ensemble learning integrates diverse features from different models and addresses their weaknesses. Image patching preserves local details and increases the dataset size. Fine-tuning allows for quick convergence and acquisition of domain-related features. Transfer learning solves the problem of limited training data. Data augmentation increases the diversity of the data and improves the generalization ability of the models, reducing the risk of overfitting. For the 6:2:2 split, the proposed ensemble model I achieved an accuracy of 99.94%, F-Score of 99.28%, precision of 99.31%, recall of 98.96%, and MCC of 99.26%. These results are significantly better than those of any of the fine-tuned models, demonstrating the efficacy of the proposed approach. In conclusion, the suggested model can aid diagnostic staff and microbiologists in the accurate identification of pathogenic bacteria, which can help control pandemics and mitigate the socioeconomic impact on society.

## Data Availability

The datasets used and/or analysed during the current study available from the corresponding author on reasonable request.
